# IL5RA as an immunogenic cell death-related predictor in progression and therapeutic response of multiple myeloma

**DOI:** 10.1038/s41598-023-35378-z

**Published:** 2023-05-26

**Authors:** Cong Xu, Meng Gao, Junhua Zhang, Yunfeng Fu

**Affiliations:** 1 Department of Hematology, The Third Xiangya Hospital of Central South University, Changsha, China; 2 Department of Blood Transfusion, The Third Xiangya Hospital of Central South University, Changsha, China

**Keywords:** Myeloma, Tumour biomarkers

## Abstract

Previous studies have shown the potential of immunogenic cell death-related modalities in myeloma. The significance of IL5RA in myeloma and immunogenic cell death remains unknown. We analyzed IL5RA expression, the gene expression profile, and secretory protein genes related to IL5RA level using GEO data. Immunogenic cell death subgroup classification was performed using the ConsensusClusterPlus and pheatmap R package. Enrichment analyses were based on GO/KEGG analysis. After IL5RA-shRNA transfection in myeloma cells, cell proliferation, apoptosis, and drug sensitivity were detected. *P* < 0.05 was considered statistically significant. IL5RA was upregulated in myeloma and progressed smoldering myeloma. We observed enrichment in pathways such as the PI3K-Akt signaling pathway, and Natural killer cell mediated cytotoxicity in the high-IL5RA group. IL5RA was also closely associated with secretory protein genes such as CST6. We observed the enrichment of cellular apoptosis and hippo signaling pathway on differential genes in the immunogenic cell death cluster. Furthermore, IL5RA was associated with immune infiltration, immunogenic cell death-related genes, immune-checkpoint-related genes, and m6A in myeloma. In vitro and in vivo experiments showed the involvement of IL5RA in apoptosis, proliferation, and drug resistance of myeloma cells. IL5RA shows the potential to be an immunogenic cell death-related predictor for myeloma.

## Introduction

Multiple myeloma (MM) is a hematological malignancy involving plasma cells with no known cure. Most patients relapse despite advances in newly developed anti-myeloma drugs such as daratumumab and elotuzumab^1^. The pathogenesis of MM consists of a long and complex evolutionary process that includes two precursor stages: monoclonal gammopathy of uncertain significance (MGUS) and smoldering MM (SMM)^2,3^. Understanding the underlying mechanisms associated with the pathogenesis of MM is of great biological and clinical interest. However, the pathogenesis is not yet fully known to date.

Immunogenic cell death (ICD) is a type of regulated cancer cell death that dying cancer cells release antigens and activate antigen-specific immune responses^4^. This process involves changes in cell surface structure and results in increased release of chemokines, proinflammatory cytokines, and pro-immunogenic factors^5^. ICD offers a new chance to increase cancer therapy effectiveness and alleviate patient suffering^6^. MM is a clonal plasma cell neoplasia with a compromised immune system. Nonetheless, immunotherapeutic therapies have the potential to be effective, as indicated by the graft-versus-myeloma effect in MM patients undergoing allogenic stem-cell transplantation or receiving donor lymphocyte infusions^7^. Hence, ICD-related modalities or the immunostimulatory potential of chemotherapeutics could be used to promote overall immunity and generate an effective immune response in MM patients^8^.

Interleukins (ILs) and interleukin receptors (ILRs) are of great significance in anti-tumor immune responses^9^. The expression or release of various interleukin family members including IL-1β, IL-6, IL-23, etc. is also involved in the process of ICD^10^. IL-5 is a highly conserved member of the IL-4 cytokine gene family that includes granulocyte/macrophage-colony-stimulating factors, IL-3, IL-4, and IL-5. Previous studies have shown that IL-5 is involved in selective eosinophilia and cell activation in allergic states^11^. At the molecular level, IL-5 promotes biological functions by recruiting a cell surface receptor consisting of two polypeptide chains, α, and β^12^. The IL-5 specific α chain, which is called IL-5 receptor α (IL-5RA), provides most of the binding energy for IL-5^13^. Previous studies have reported the potential of IL-5RA as a predictor for drug resistance, prognosis, and immunotherapy response in cancer^14,15^. However, the significance of IL-5RA in MM remains unknown.

In this study, we investigated the expression of IL5RA in MM and SMM using GEO data. Then, analysis of secretory protein (SP) profile related to IL5RA level, ICD subgroup, and correlations between IL5RA and ICD, immune infiltration, immune-checkpoint, or N6-methyladenosine (m6A) related genes in MM were conducted. Finally, the function of IL5RA in MM was preliminarily explored by in vitro and in vivo experiments. Our study reveals for the first time the association of differentially expressed IL5RA with ICD and its potential as a predictor for therapeutic response in MM.

## Methods

### Differential expression analysis

We downloaded GSE125361 (n = 48) microarray data from the Gene Expression Omnibus (GEO) database, which included 45 myeloma samples and 3 controls, for expression analysis of IL5RA in cancer^16^. Additionally, we analyzed the expression of IL5RA in smoldering myeloma (SMM) patients who progressed to active MM (n = 10) and non-progressed SMM (at a minimum of 36 months, n = 12) using the GSE117847 dataset by GEO2R.

### Analysis of secretory protein profile related to IL5RA

Firstly, we analyzed the gene expression profiling related to IL5RA in GSE125361. Tumors were divided into high- and low-IL5RA expression groups according to the median value, and differentially expressed genes (DEGs) were analyzed using the limma package (v3.40.2) in the R software. “Adjusted *P* < 0.05 and log fold change (logFC) > 1 or logFC < − 1” were defined as the thresholds for a statistical difference. The results of the differential analysis were displayed in volcano plots and heat maps. To further confirm the underlying function, DEGs were enriched using Gene Ontology (GO) and Kyoto Encyclopedia of Genes and Genomes (KEGG) enrichment analysis^17^.

Then, we used the Human Protein Atlas (HPA) protein annotation database to download the secretory protein list and the Uniprot database to download the extracellular protein list GO:0005615^18,19^. The two lists were intersected with DEGs and the union results were taken as SP-DEGs. Visualization of enrichment analysis of SP-DEGs was performed using the GOplot (v1.0.2) and ggplot2 (v3.3.3) packages in R.

### ICD subgroup classification

Microarray data from GSE24080, which contained more than 500 MM samples, was downloaded from the GEO database. Consistency analysis was performed using ConsensusClusterPlus R package (v1.54.0), the number of clusters was 2, and 80% of the total sample was drawn 100 times, innerLinkage = ‘ward.D2’, clusterAlg = “hc”. The R software package pheatmap (v1.0.12) was used for clustering heatmaps. The gene expression heatmap retained genes with square deviation > 0.1 if the input genes number was less than 1000. Otherwise, it will extract the top 25% of genes after sorting their square deviation. Differentially expressed genes analysis of two ICD clusters was performed as previously described. Then, we analyzed the functions of the differential genes by GO and KEGG enrichment.

### Gene expression correlation analysis

Spearman’s rank correlation analysis was performed to assess correlations between IL5RA and ICD, immune-checkpoint, or m6A-related genes in GSE24080. ICD-related genes ENTPD1, NT5E, CALR, HMGB1, HSP90AA1, ATG5, BAX, CASP8, PDIA3, EIF2AK3, PIK3CA, CXCR3, IFNA1, IFNB1, IL10, IL6, TNF, CASP1, IL1R1, IL1B, NLRP3, P2RX7, LY96, MYD88, TLR4, CD4, CD8A, CD8B, FOXP3, IFNG, IFNGR1, IL17A, IL17RA, and PRF1 summarized by Abhishek et al.^20^. Common immune checkpoints include CD274, CTLA4, HAVCR2, LAG3, PDCD1, PDCD1LG2, SIGLEC15, and TIGIT^21^. The R software package ggplot2 (v3.3.3) was used to plot scatter plots, correlation heat maps, lollipop diagrams, and calculate correlation coefficients. The chord diagram was drawn in R using the circlize package (v0.4.12)^22^. For group-wise comparisons, samples were divided into high- and low- IL5RA expression groups as previously described.

### Immune infiltration analysis

We analyzed the immune infiltration associated with IL5RA expression in the GSE24080 dataset using the quantiseq algorithm from the immunedeconv package (v2.1.0). The infiltration levels of 10 common immune cells in the IL5RA low or high expression group were compared by the Wilcoxon rank sum test and visualized by ggplot2 (v3.3.3).

### Clinical tissues and cell culture

We obtained bone marrow specimens from iron-deficiency anemia (taken as normal control), SMM, and MM patients at the Third Xiangya Hospital. All patients provided their written informed consent. Research on clinical samples was performed in accordance with the Declaration of Helsinki. All MM and SMM patients met the diagnostic criteria defined by National Comprehensive Cancer Network (NCCN). Human MM cell lines (ARP1 and RPMI-8226) were purchased from the National Collection of Authenticated Cell Cultures (China). Cells were cultured in 5% CO_2_ at 37  °C with RPMI-1640 medium (HyClone, USA) plus 10% fetal bovine serum (ExCell Biology, Shanghai, China) and 1% penicillin–streptomycin (HyClone, USA). The shRNA-IL5RA and shRNA-NC were synthesized by GenePharma^23^. The cells were transfected using Lipofectamine 2000 (Invitrogen, USA) according to the manufacturer’s protocol. For the cell proliferation assay, myeloma cells were incubated in 96-well plates for the required time. Then, cell count kit-8 (CCK-8) reagent (Sigma-Aldrich, St Louis, Missouri; 10 µL) was added followed by incubation for 4 h. The absorbance of the solution was measured at 450 nm with a microplate imaging system.

### RNA extraction and qPCR

Firstly, we used anti-CD138 MicroBeads (Miltenyi, Germany) to enrich plasma cells from bone marrow samples. The purity of separated plasma cells met above 90% (identified by flow cytometry). Then, we used the standard TRIzol (Invitrogen, United States) RNA extraction protocol to extract total RNA from control or plasma cell samples. Prepared RNA samples were preserved at − 80 °C. All the RNA samples were quantified with NanoDrop ND-1000 (NanoDrop, USA). Reverse transcription was conducted using PrimeScript RT Reagent Kit (Takara, China). Real-time qPCR was performed using Brilliant II SYBR Green RT–qPCR kit. We used the 2^−ΔΔCt^ method to calculate relative levels of IL5RA. The primers used are shown as follows: for IL5RA, F: 5′-TGAAAGAGTGAAGAACCGCC-3′, R: 5′-CCTGGCCTGAGAAATGCG-3′, for GAPDH: F: 5′-CTCTGCTCCTCCTGTTCGAC-3′, R: 5′-ACGACCAAATCCGTTGACTC-3′^24^.

### Tumor xenografts in mice

Animal experiments were conducted following the protocol approved by the Institutional Animal Care and Ethics Committee of Central South University, China. Animal experiments followed the recommendations in the ARRIVE guidelines. We established a subcutaneous MM tumor model in NOD/SCID immunodeficient mice (Biocytogen, China). To be more specific, mice were subcutaneously inoculated with ARP1 MM cell line transduced with IL5RA-shRNA or scramble-shRNA (1 × 10^6^ cells in 100 μL medium) into the left abdomen. Tumor volume was recorded to track tumor burden. The mice were euthanized when tumor diameters reached approximately 1.5 cm or a humane endpoint was reached.

### Immunofluorescence and immunohistochemistry

Tissues from mice were dissected, fixed in 10% buffered formalin, and paraffin-embedded. Tissue sections were deparaffinized with Histoclear before antigen retrieval in citrate buffer at pH 6.4. The activity of endogenous peroxidase (HRP) was inhibited by treating the sections with 3% hydrogen peroxide. In the presence of avidin-horseradish peroxidase and DAB color substrate, immunohistochemistry or immunofluorescence was performed using antibodies or fluorescent antibodies, followed by species-specific biotinylated secondary antibodies. Tissue sections were counterstained with hematoxylin or DAPI after immunohistochemistry or immunohistochemistry. The slides were then photographed and scanned.

### Statistical analyses

All statistical analyses were performed and visualized based on R (v4.0.3). Statistical significance was assessed using two-sided student t-tests for data that obeyed normal distribution. Otherwise, data were evaluated using the Wilcoxon rank sum test. The receiver operating characteristic (ROC) curve was used to explore the possibility of IL5RA as a diagnostic marker. *P* < 0.05 was considered statistically significant.

## Results

### IL5RA expression was upregulated in MM and progressed-SMM

**Figure 1:**
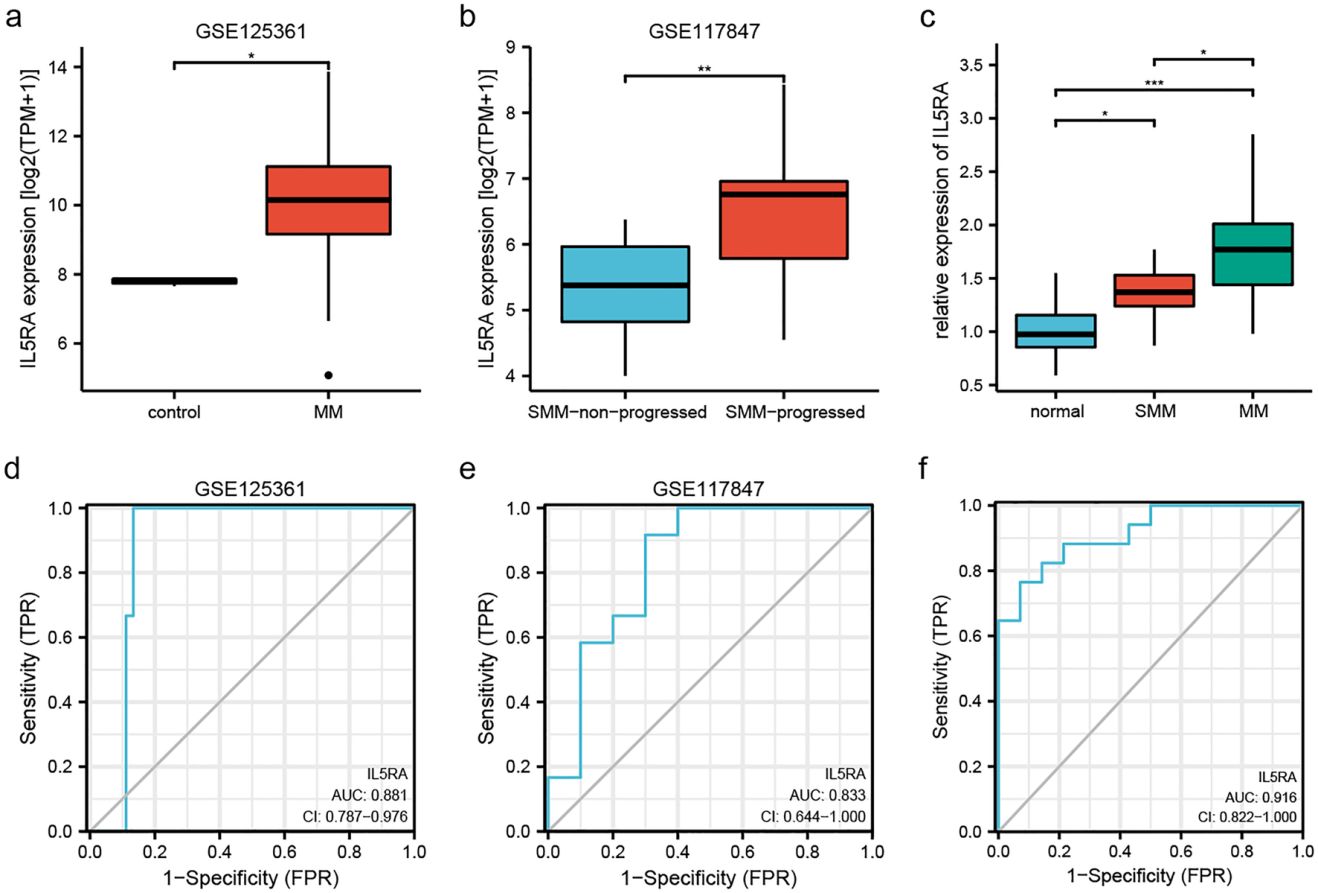
IL5RA expression in MM and progressed-SMM. (**a**) IL5RA expression was significantly upregulated in MM compared to normal controls. (**b**) In SMM patients who progressed to active MM, IL5RA expression was upregulated. (**c**) IL5RA expression was significantly upregulated in MM and SMM in clinical samples. (**d**) Diagnostic value of IL5RA in MM in GSE125361. (**e**) Diagnostic value of IL5RA in progressed-SMM in GSE117847. (**f**) Diagnostic value of IL5RA in MM in clinical samples.

First, we explored the expression of IL5RA in plasma cell disease using the GEO database. As shown in Fig. 1a, IL5RA was significantly elevated in MM compared to normal controls (the GSE125361 series matrix was available in supplementary Table 1). In SMM, the subgroup that eventually progresses to MM had higher IL5RA expression (Fig. 1b) (the GSE117847 series matrix was available in supplementary Table 2). Besides, we collected bone marrow samples from 14 controls, 15 SMM, and 17 newly diagnosed MM patients. The qPCR results showed that IL5RA was upregulated in SMM and MM patients compared with controls, and the IL5RA level in MM patients was significantly higher than that in SMM (Fig. 1c). We also evaluated the diagnostic performance of IL5RA in MM or progressive SMM. IL5RA demonstrated an AUC of 0.881 (95% CI 0.787–0.976) in being able to differentiate MM patients from normal controls (Fig. 1d). IL5RA also showed good diagnostic accuracy in progressed-SMM with an AUC of 0.833 (95% CI 0.644–1.000) (Fig. 1e). The diagnostic significance of IL5RA for MM was confirmed in clinical samples (AUC 0.916, 95% CI 0.822–1.000) (Fig. 1f).

### Whole gene expression profile and secretory protein genes related to IL5RA level

**Figure 2:**
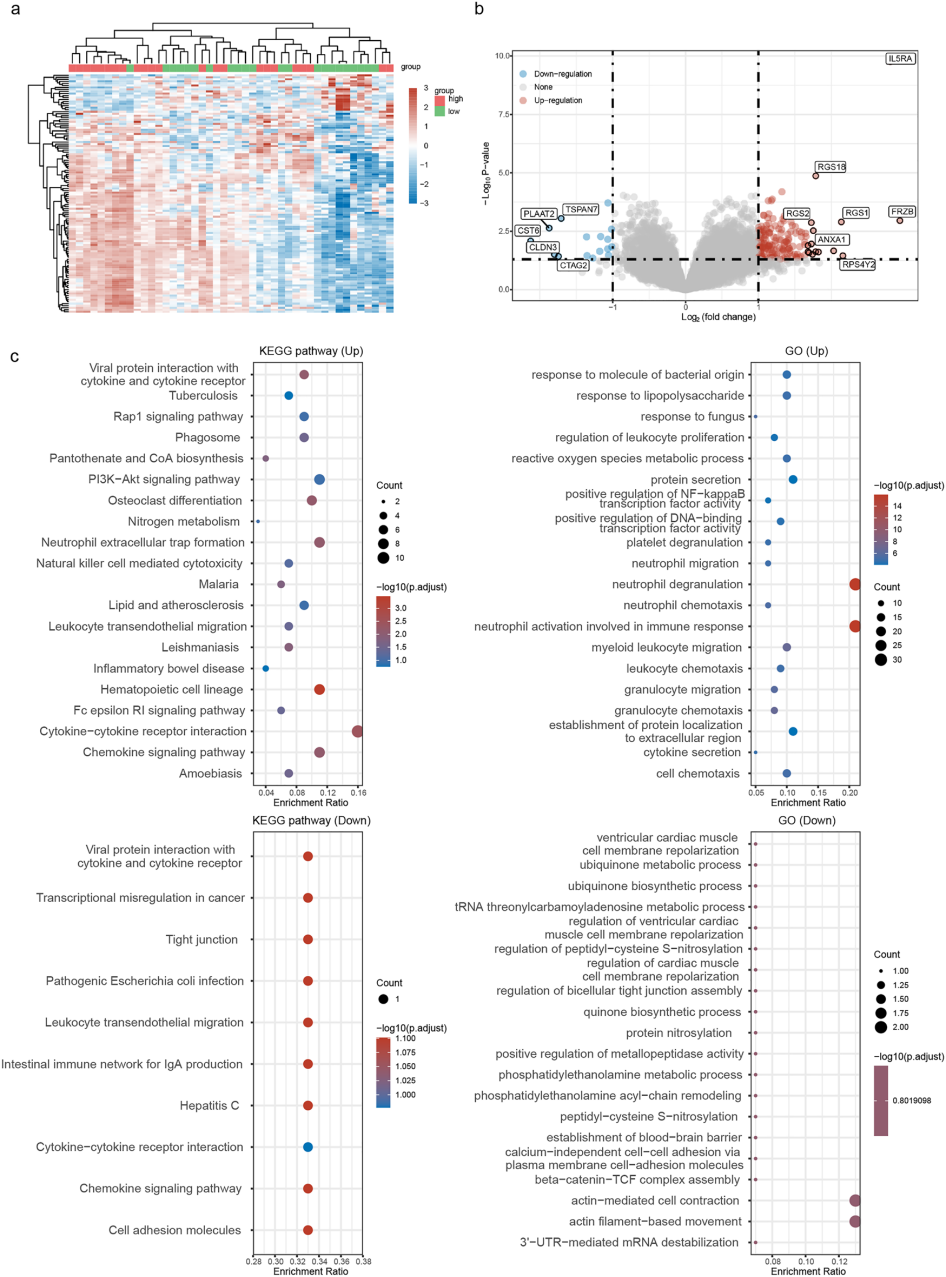
Differential whole gene profiling related to IL5RA expression. (**a**) Heat map of differentially expressed gene profiling based on IL5RA status. (**b**) The volcanic plot showed 158 upregulated genes and 18 downregulated genes related to IL5RA expression. (**c**) GO enrichment and KEGG pathway analysis on differentially expressed genes related to IL5RA level.

Analysis of differentially expressed gene profiling related to IL5RA was performed to further study the biological function of IL5RA in MM. A total of 158 upregulated genes and 18 downregulated genes were considered to be significantly related to IL5RA expression (Fig. 2a,b) (Complete list of DEGs was available in supplementary Table 3). Then, we performed GO enrichment and KEGG pathway analysis on DEGs. These genes were uniquely enriched in specific pathways, with the enrichment of the Chemokine signaling pathway, Cytokine-cytokine receptor interaction, PI3K-Akt signaling pathway, Rap1 signaling pathway, Natural killer cell mediated cytotoxicity, which suggest the involvement in the regulation of tumor, signal transduction, and cellular immunity (Fig. 2c).

**Figure 3:**
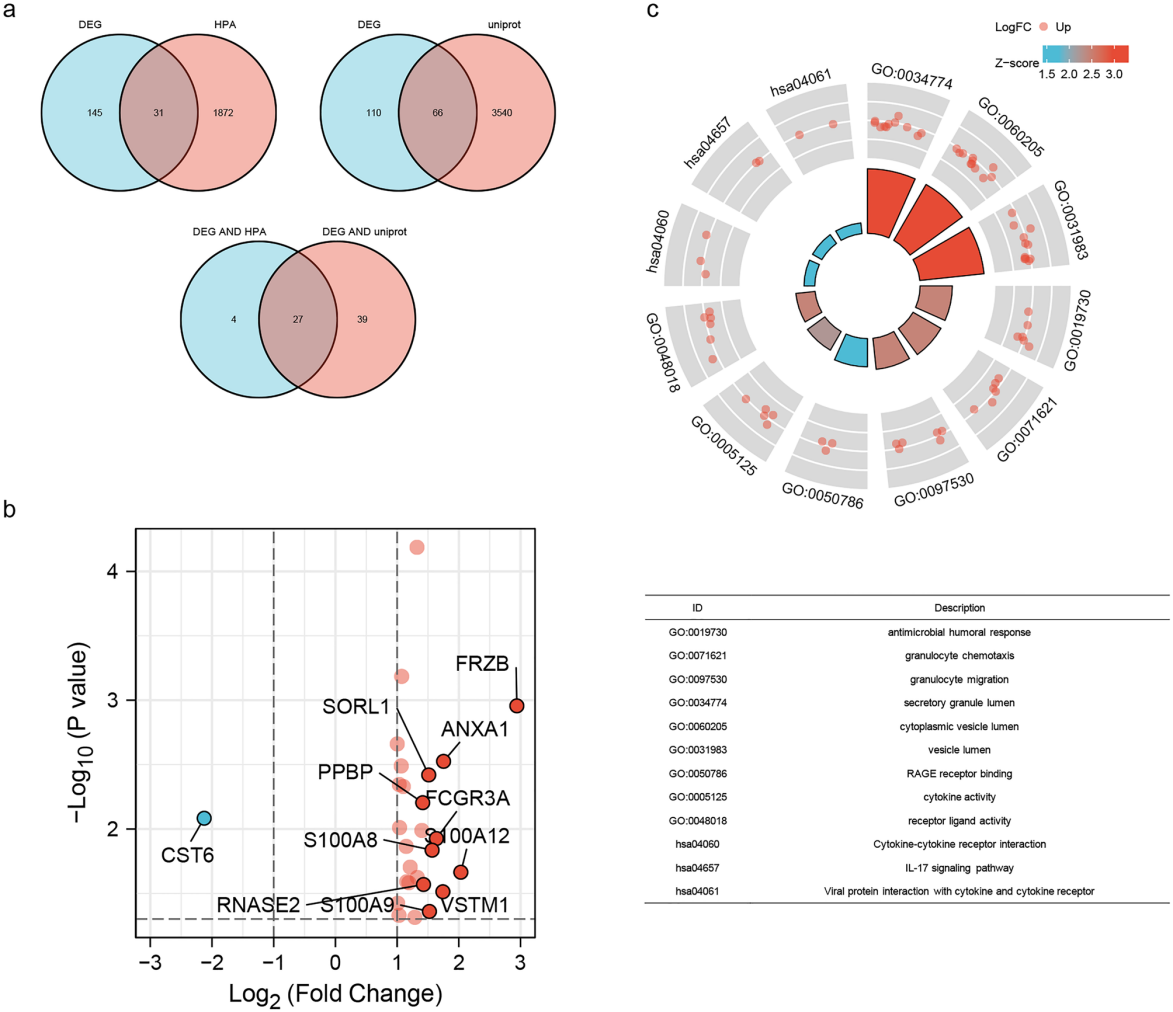
Secretory protein profile associated with IL5RA expression. (**a**) Screening for secretory protein genes associated with IL5RA. (**b**) Volcanic map of SP-DEGs related to IL5RA. (**c**) GO/KEGG enrichment analysis of SP-DEGs.

We further analyzed the differences in secretory protein profiles associated with IL5RA expression. Secretory protein genes annotated in the Human Protein Atlas (HPA) database were intersected with DEGs, and 31 SP-DEGs were identified. Secretory protein genes annotated in the Uniprot database were intersected with DEGs, yielding 66 SP-DEGs. The SP-DEGs obtained by these two methods were combined, yielding a total of 27 SP-DEGs, 26 of which were up-regulated and one of which was down-regulated (Fig. 3a,b). GO and KEGG enrichment analysis showed that SP-DEGs were mainly enriched in the antimicrobial humoral response, granulocyte chemotaxis, granulocyte migration, secretory granule lumen, cytoplasmic vesicle lumen, vesicle lumen, RAGE receptor binding, cytokine activity, receptor ligand activity, Cytokine-cytokine receptor interaction, IL-17 signaling pathway, Viral protein interaction with cytokine and cytokine receptor (Fig. 3c).

### Relationship between ICD cluster and whole gene expression profile

**Figure 4:**
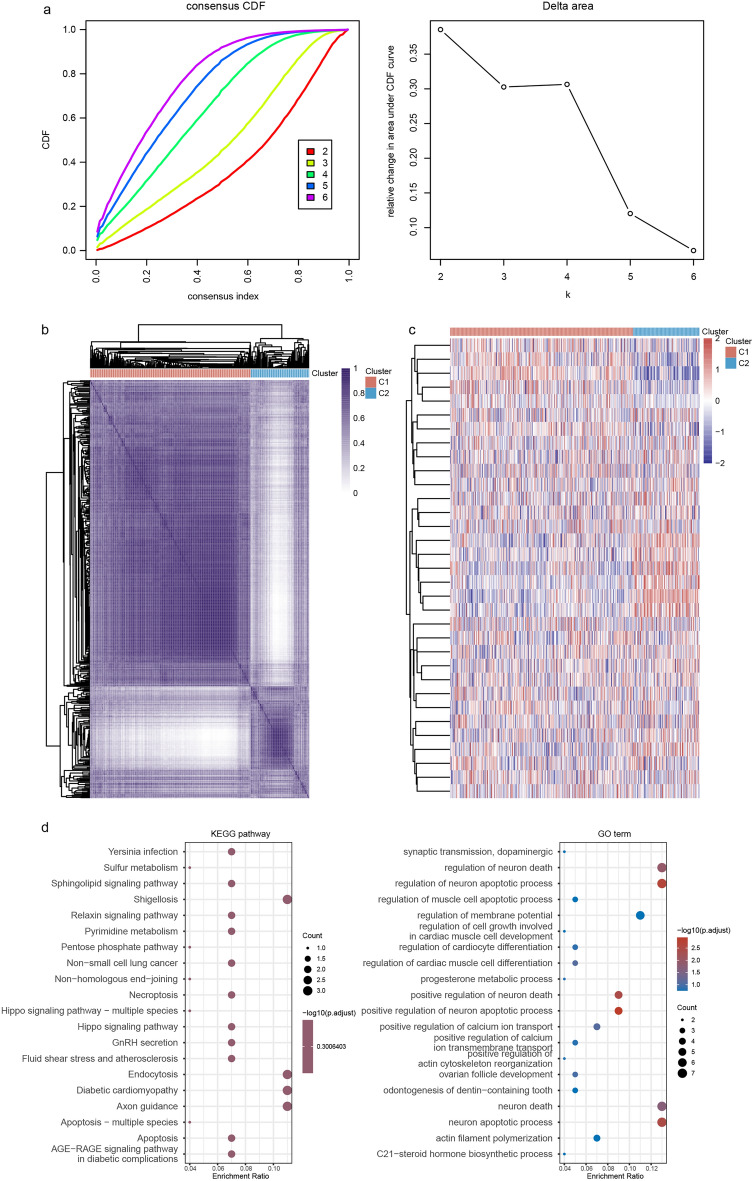
Relationship between ICD cluster and whole gene expression profile in MM. (**a**) Consensus clustering CDF and relative changes in the CDF Delta area. The abscissa indicates the category number k, and the ordinate indicates the relative change in the area. (**b**) Heatmap of consistency of clustering results. Rows and columns represent different samples, the different colors represent different types. (**c**) The expression heatmap of ICD genes in different clusters. (**d**) KEGG and GO enrichment analysis on differentially expressed genes in ICD clusters in MM.

The GSE24080 MM dataset was grouped in cluster-1 (C1) and cluster-2 (C-2) based on the ICD-associated gene set. Figure 4a presents the consensus clustering cumulative distribution function (CDF) and relative change in the area under the CDF curve (CDF Delta area). Figure 4b and c demonstrate the consistency of the clustering results heatmap and the expression heatmap of ICD genes in different subgroups, respectively. We analyzed differentially expressed genes in C1 and C2 groups and further performed KEGG and GO enrichment analysis. The enrichment results suggested that the differences in the expression levels of ICD-related genes may be related to ion transmembrane transport, cellular apoptosis, and the hippo signaling pathway in MM (Fig. 4d).

### IL5RA was associated with ICD, immune infiltration, and immunotherapy

**Figure 5:**
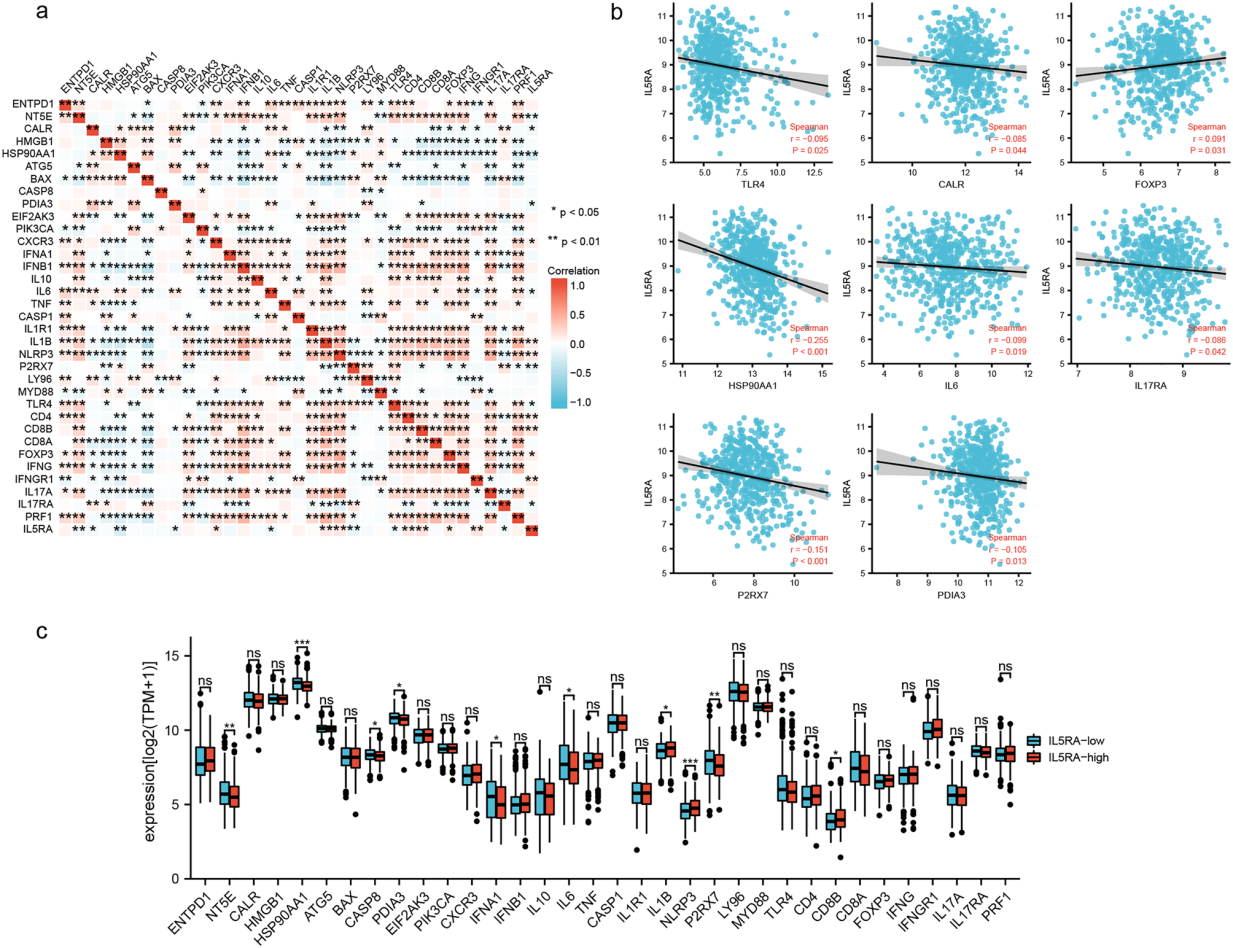
Correlation between IL5RA and ICD-related genes in MM. (**a**) Correlation heat map of IL5RA and ICD-related genes in MM. (**b**) Scatter plots of the correlation between IL5RA and ICD-related genes. ICD-related genes include TLR4, CALR, HSP90AA1, IL6, IL17RA, P2RX7, PDIA3. (**c**) Variation among ICD-related genes in the high and low IL5RA expression groups in MM.

We examined the expression correlation of 34 ICD-related genes and IL5RA in GSE24080. As shown in Fig. 5a, expression levels of 34 ICD-related genes were closely correlated in MM. In addition, IL5RA was associated with the expression of 15 different ICD-related genes. In general, highly expressed ICD-related genes were associated with good clinical prognosis, except for ENTPD1, NT5E, IL10, and FOXP3. In our study, IL5RA was negatively correlated with TLR4, CALR, HSP90AA1, IL6, IL17RA, P2RX7, PDIA3 expression levels, and positively correlated with FOXP3 expression (Fig. 5b). This suggests that IL5RA may regulate the progression of MM by mediating ICD. We also analyzed the differential expression of ICD-related genes between different IL5RA-level groups in MM. Compared with the low IL5RA expression group, the expression of HSP90AA1, CASP8, PDIA3, IFNA1, IL6, and P2RX7 were significantly downregulated in the high IL5RA expression group (Fig. 5c).

**Figure 6:**
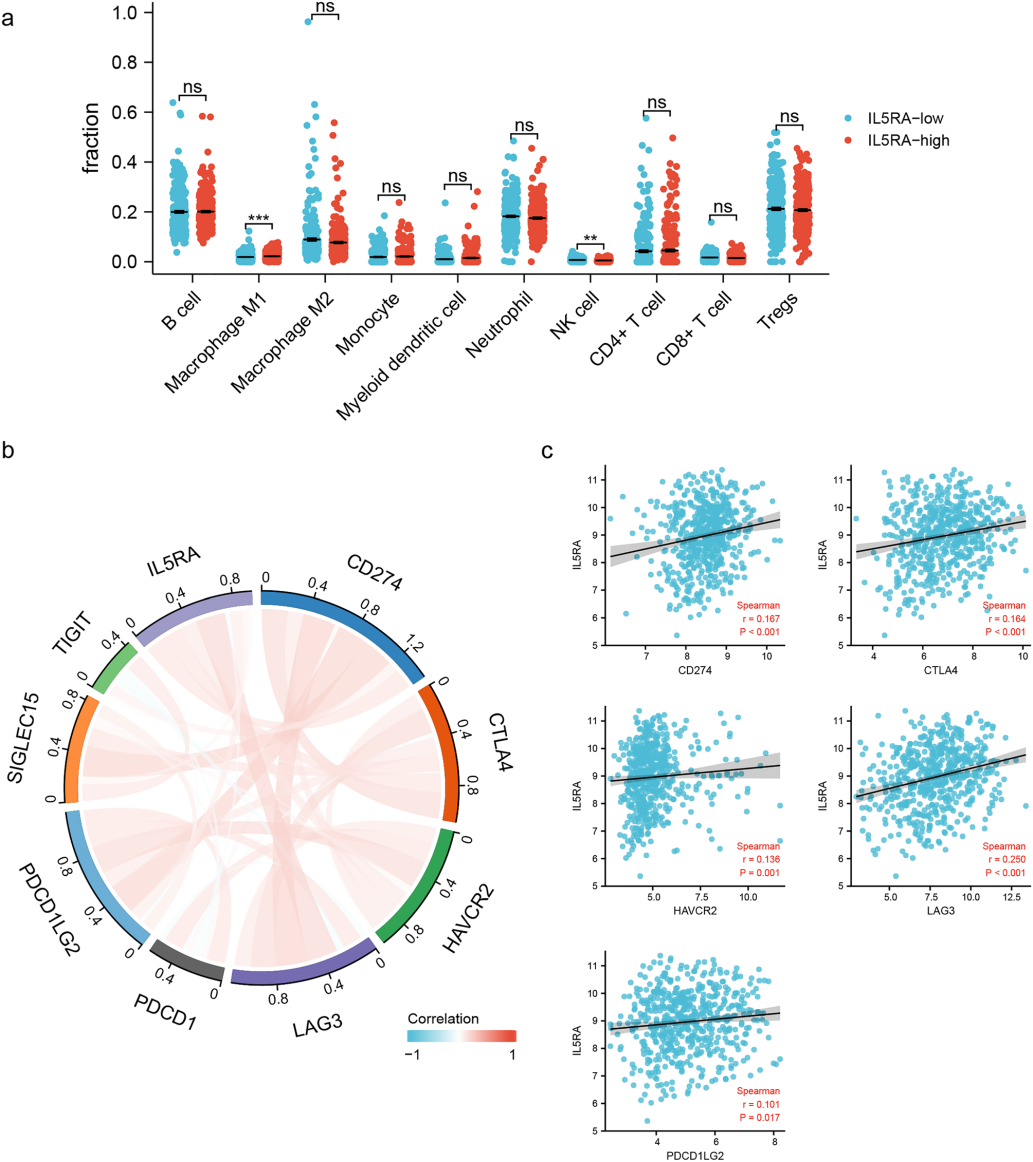
IL5RA was associated with immune infiltration and immune checkpoints in MM. (**a**) IL5RA-associated immune infiltration in MM. (**b**) Chord diagram of IL5RA and immune-checkpoint-related genes in MM. (**c**) Scatter plots of the correlation between IL5RA and CD274, CTLA4, HAVCR2, LAG3, and PDCD1LG2.

We analyzed the correlation between IL5RA and immune cell infiltration in MM. As shown in Fig. 6a, IL5RA was closely related to the cell infiltration level of Macrophage M1 and NK. We further analyzed immune checkpoint gene expression and its relevance to IL5RA in MM. As shown in Fig. 6b, the expressions of almost all immune checkpoint genes in MM positively correlated. Further analysis showed a positive correlation between the level of IL5RA and CD274, CTLA4, HAVCR2, LAG3, or PDCD1LG2 in MM (Fig. 6c).

### IL5RA was associated with m6A RNA methylation regulators

**Figure 7:**
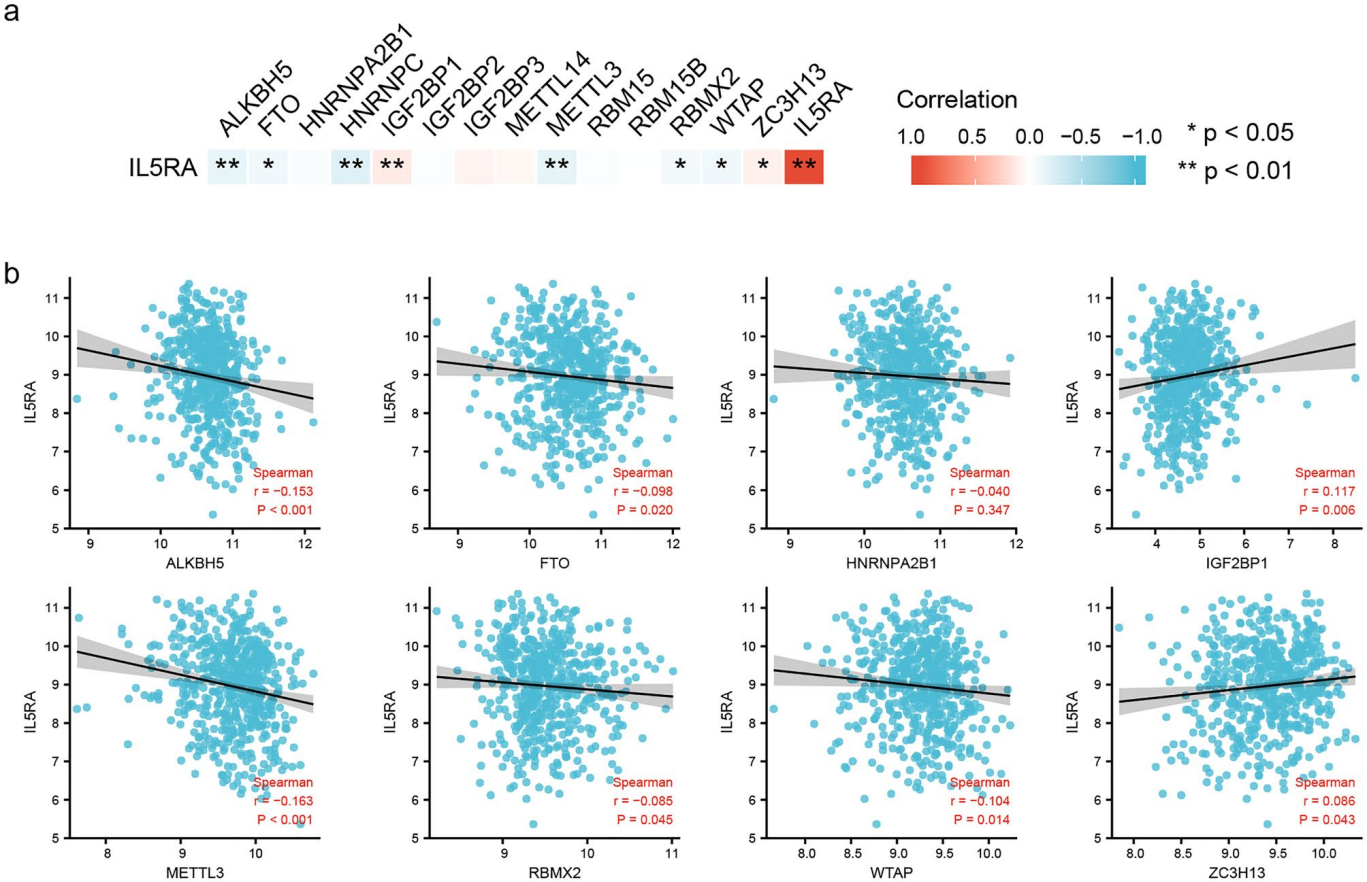
IL5RA was associated with m6A genes in MM. (**a**) Correlation between the expression of IL5RA and m6A-related genes. (**b**) Scatter plots of the correlation between IL5RA and ALKBH5, FTO, HNRNPA2B1, METTL3, RBMX2, WTAP, IGF2BP1 and ZC3H13.

M6A modifications have been identified to be involved in tumor immunity, proliferation, angiogenesis, metastasis, and other processes^25^. We analyzed the correlation between the expression of IL5RA and 15 m6A-related genes (some m6A-related gene data were missing in GSE24080) (Fig. 7a). As shown in Fig. 7b, the IL5RA expression was significantly negatively correlated with ALKBH5, FTO, HNRNPA2B1, METTL3, RBMX2, WTAP and positively correlated with IGF2BP1, ZC3H13.

### Regulation of IL5RA on proliferation, apoptosis, and drug sensitivity of MM cells

**Figure 8:**
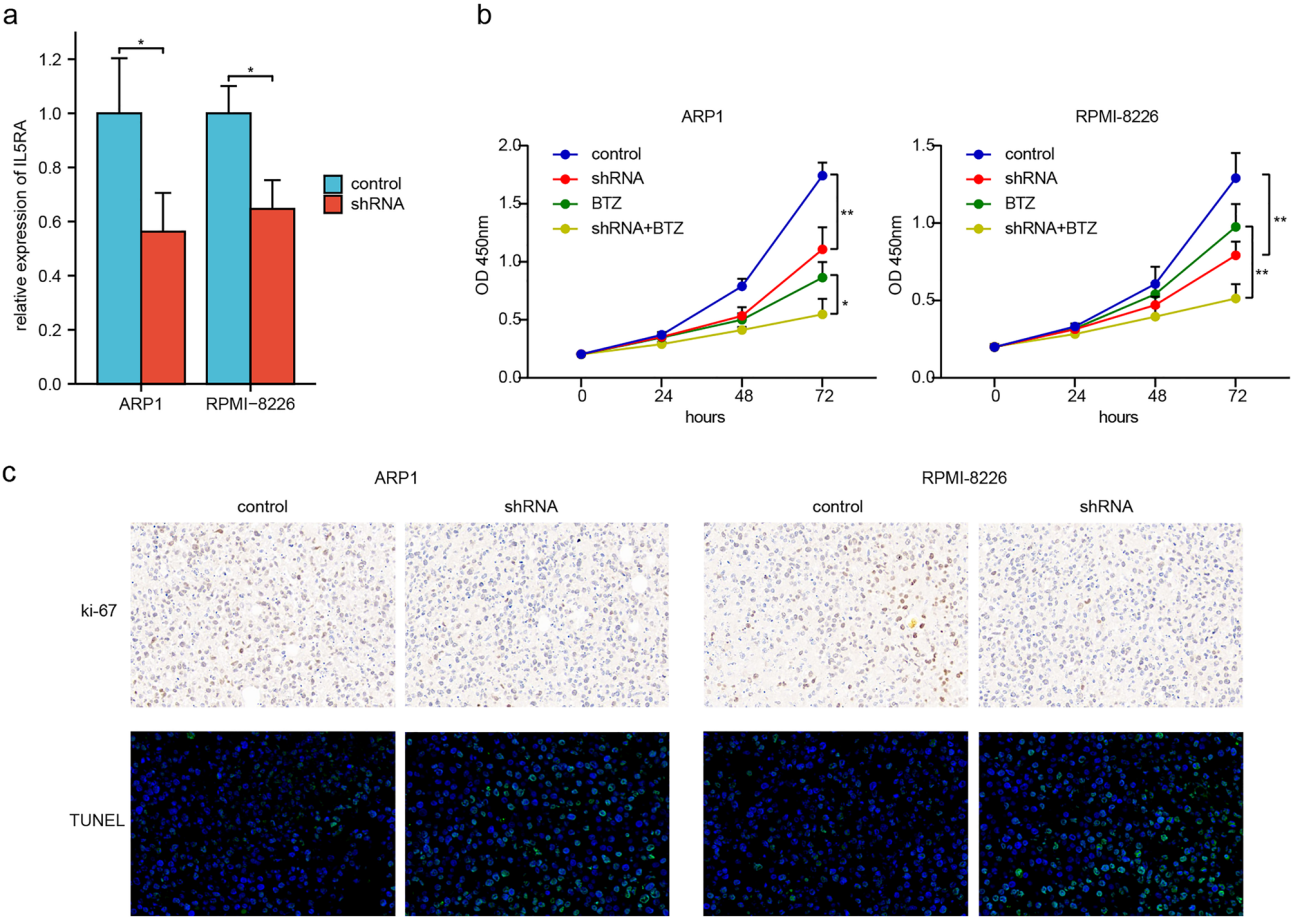
IL5RA function in vivo and in vitro. (**a**) IL5RA shRNA inhibition efficiency. (**b**) CCK-8 was used to detect the regulation of IL5RA on cell proliferation and BTZ (5 nM) drug sensitivity in MM cells. (**c**) The subcutaneous MM tumor samples were collected, then ki-67 was detected by immunohistochemistry and TUNEL was detected by immunofluorescence.

We validated the function of IL5RA in vitro and in vivo. IL5RA expression was inhibited by shRNA in ARP1 and RPMI-8226 cells (Fig. 8a). Inhibition of IL5RA resulted in decreased proliferation and increased sensitivity to BTZ in vitro (Fig. 8b). Finally, we examined the effect of IL5RA on MM proliferation and apoptosis in vivo. Inhibition of IL5RA resulted in decreased ki-67 index and an increased proportion of TUNEL-positive cells (Fig. 8c).

## Discussion

Dysregulation of oncogenes and tumor suppressor genes are the main features of cancer. The development and progression of plasma cell disease are complex processes that still needs further clarification. Previous studies have shown the role of the interleukin family in the pathogenesis, therapeutics, and bone disease of MM^26–28^. For IL5RA, Fan et al. reported its potential as a predictor of prognosis and immunotherapy response in lung cancer, and Martin et al. showed its upregulation in drug-resistant Hodgkin lymphoma cells^14,15^. Our study revealed for the first time that IL5RA was highly expressed in MM and SMM patients that progressed to active MM. The enrichment analysis of DEGs related to IL5RA showed enrichment in cancer-related pathways. On the other hand, myeloma cells exhibit a high protein secretory load, which has been considered to be closely related to osteoclast signaling in MM^29,30^. Our study shows that IL5RA is associated with secretory proteins such as CST6, while CST6 has been found to suppress osteolytic bone disease in MM by blocking osteoclast differentiation^31^. In summary, these suggest that IL5RA may be involved in the progression of plasma cell tumors.

Over the past years, treatment options and survival rates of MM patients have undergone a radical improvement, with monoclonal antibodies, proteasome inhibitors, immunomodulatory drugs, and autologous stem-cell transplantation as the contributors to this advance^32^. However, drug resistance is still the main concern and accounts for the fatality of most patients^33^. That is why novel immunotherapeutic approaches may be more effective. Currently, immunotherapeutic options include antibody-based therapy, adoptive cell therapy, releasing the brakes with checkpoint blockade and DC-based vaccines (and their enhancement/upgrade with ICD) are providing positive results in relapsed and refractory MM patients. The correlation of IL5RA with immune checkpoint gene expression suggests its potential to mediate immunotherapy in MM. Noteworthy, these immunotherapeutic approaches may theoretically be benefited from ICD^10^. The landscape of ICD-related gene expression in MM remains largely unknown to date. Our study suggested a close correlation between the expression levels of more than 30 ICD-related genes in MM. Analysis of ICD clusters also suggested gene enrichment in transmembrane transport, cellular apoptosis, and cancer-related signaling pathways. This suggests the potential significance of ICD-related genes in MM. In addition, as mentioned above, high expression of most ICD-related genes is associated with a good prognosis^20^. Our study showed that IL5RA was negatively related to ‘good’ ICD-related genes and positively related to ‘poor’ ICD-related genes in MM. Further in vitro and in vivo experiments showed that IL5RA is involved in the regulation of proliferation and drug resistance of MM cells. These suggest the potential of IL5RA to mediate MM progression and drug responsiveness by regulating ICD.

The immune infiltrates are closely associated with the survival and response to immune therapy in cancer patients. Recent studies have shown the great significance of the features of immune infiltration in MM^34,35^. On the other hand, although the association between IL5RA and immune infiltration in MM remains unknown, studies have shown that the IL5RA-based model presents unique characteristics in immune infiltration in the tumor microenvironment and exhibits the potential as a biomarker for predicting immunotherapy response in lung adenocarcinoma^14^. Our study showed that the expression level of IL5RA was closely related to immune infiltrates and immune checkpoints in MM. These findings suggest that IL5RA may be a predictor for immunotherapy response in MM.

As the most abundant internal modification in eukaryotic mRNA, m6A modification and the associated regulatory proteins have been revealed to play critical roles in cancer biology^36^. m6A modifications also play important roles in cancer immunity. For example, the inhibition of METTL3 in dendritic cells leads to a decrease in the levels of IL-12 and the costimulatory molecules CD40, CD80, and weakens T-cell activation in vitro^37^. In colorectal cancer, the reduction of METTL3 or METTL14 rises cytotoxic tumor-infiltrating CD8+ T cells and stimulates the recruitment of CD4+ and CD8+ effector T cells that suppress tumor growth^38^. Currently, the correlation between IL5RA and m6A modification remains unknown. In the present study, we found significant associations between IL5RA and multiple m6A genes in MM. These indicate that IL5RA may mediate MM immune via m6A regulation.

In conclusion, we identify that IL5RA is highly expressed in MM and high-risk SMM (progressing to MM). IL5RA may be involved in cell signal transduction, immune infiltration, immune checkpoints, and m6A modification in MM. In particular, our study reveals the landscape of ICD expression and the potential regulatory significance of IL5RA on ICD in MM. IL5RA may be a potential ICD-related predictor for therapeutic response in MM.

## Supplementary Information

Supplementary Table 1.

Supplementary Table 2.

Supplementary Table 3.

## Data Availability

All gene expression profile data were obtained from the GEO public database (https://www.ncbi.nlm.nih.gov/geo/query/acc.cgi?acc=GSE125361, https://www.ncbi.nlm.nih.gov/geo/query/acc.cgi?acc=GSE117847, https://www.ncbi.nlm.nih.gov/geo/query/acc.cgi?acc=GSE24080). Other original contributions presented in the study are included in the article. Further inquiries can be directed to the corresponding author.
